# m^5^C RNA methylation: a potential mechanism for infectious Alzheimer’s disease

**DOI:** 10.3389/fcell.2024.1440143

**Published:** 2024-08-08

**Authors:** Sisi Teng, Cunqiao Han, Jian Zhou, Zhenyan He, Weiwei Qian

**Affiliations:** ^1^ Department of Neurology, Shangjinnanfu Hospital, West China Hospital, Sichuan University, Chengdu, Sichuan, China; ^2^ Department of Emergency, Shangjinnanfu Hospital, West China Hospital, Sichuan University, Chengdu, Sichuan, China; ^3^ Department of Immunology, International Cancer Center, Shenzhen University Health Science Center, Shenzhen, Guangdong, China; ^4^ National-Regional Key Technology Engineering Laboratory for Medical Ultrasound, School of Biomedical Engineering, Shenzhen University Medical School, Shenzhen, Guangdong, China; ^5^ Department of Neurosurgery, The Affiliated Cancer Hospital of Zhengzhou University and Henan Cancer Hospital, Zhengzhou, China; ^6^ Department of Emergency Medicine, Laboratory of Emergency Medicine, West China Hospital, and Disaster Medical Center, Sichuan University, Chengdu, Sichuan, China

**Keywords:** RNA methylation, m5C methylation, Alzheimer’s disease, infectious etiology, neurodegenerative disorder

## Abstract

Alzheimer’s disease (AD) is a neurodegenerative disorder caused by a variety of factors, including age, genetic susceptibility, cardiovascular disease, traumatic brain injury, and environmental factors. The pathogenesis of AD is largely associated with the overproduction and accumulation of amyloid-β peptides and the hyperphosphorylation of tau protein in the brain. Recent studies have identified the presence of diverse pathogens, including viruses, bacteria, and parasites, in the tissues of AD patients, underscoring the critical role of central nervous system infections in inducing pathological changes associated with AD. Nevertheless, it remains unestablished about the specific mechanism by which infections lead to the occurrence of AD. As an important post-transcriptional RNA modification, RNA 5-methylcytosine (m^5^C) methylation regulates a wide range of biological processes, including RNA splicing, nuclear export, stability, and translation, therefore affecting cellular function. Moreover, it has been recently demonstrated that multiple pathogenic microbial infections are associated with the m^5^C methylation of the host. However, the role of m^5^C methylation in infectious AD is still uncertain. Therefore, this review discusses the mechanisms of pathogen-induced AD and summarizes research on the molecular mechanisms of m^5^C methylation in infectious AD, thereby providing new insight into exploring the mechanism underlying infectious AD.

## 1 Introduction

Alzheimer’s disease (AD) is a complex neurodegenerative disorder typically manifesting as memory loss, cognitive decline, and behavioral abnormalities. Although its exact etiology has not yet been fully clarified, AD has been strongly associated with a multitude of factors, including age, genetic predisposition, cardiovascular disease, traumatic brain injury, and environmental factors (Lane et al., 2018; Soria Lopez et al., 2019; Graff-Radford et al., 2021; Scheltens et al., 2021; Author Anonymous, 2023). Available studies of AD have centered on the overproduction and accumulation of amyloid-β (Aβ) peptides and the hyperphosphorylation of tau protein in the brain (Brody, 2011; Mantzavinos and Alexiou, 2017; Rostagno, 2022). Additionally, the pivotal role of infections in the etiology of AD is gaining widespread attention and being intensively studied because of increasing relevant evidence (Lim et al., 2015; Eimer et al., 2018; Mancuso et al., 2019; Huang et al., 2021a; Piekut et al., 2022; Baranova et al., 2023).

Although the infectious etiology of AD was first proposed back in 1907, it has not been widely accepted for a long time (Woods et al., 2020). Of note, advances in modern research techniques have enabled deeper investigation into the potential role of infections in AD. Numerous studies have unveiled that various pathogens are involved in the pathological process of AD via direct or indirect mechanisms. For example, cognitive decline in AD is also associated with viruses such as herpes simplex virus (HSV-1, 2, 6A/B), human cytomegalovirus, Epstein-Barr virus, hepatitis C virus, influenza virus, and severe acute respiratory syndrome coronavirus 2 (SARS-CoV-2), bacteria (including *T. pallidum*, *B. burgdorferi*, *C. pneumoniae*, *P. gingivalis*, *P. intermedia*, *Tannerella forsythia*, *F. nucleatum*, *Aggregatibacter actinomycetemcomitans*, *E. corrodens*, *Treponema denticola*, and *H. pylori*), and even some parasites (*T. gondii*) in eukaryotic single cells (Sochocka et al., 2017; Piekut et al., 2022). These pathogens are involved in the pathological changes of AD by regulating multiple pathways. Viral and bacterial infections may trigger chronic inflammatory responses, leading to neuronal damage and Aβ peptide overproduction (Piekut et al., 2022). Aβ peptides have long been considered to predominantly have a contributing role to the development of AD since they form neurotoxic plaques that compromise neuronal function when over-accumulated in the brain of AD patients (O’Brien and Wong, 2011; Tiwari et al., 2019). Nevertheless, recent studies have demonstrated that Aβ peptides are not entirely detrimental and also have a spectrum of protective effects in the body. For instance, Aβ peptides can exert antimicrobial activity, protect against infections, repair leaks in the blood-brain barrier (BBB), promote recovery from brain injury, and modulate synaptic function (Eimer et al., 2018; Chen et al., 2022; Shi et al., 2022). Therefore, Aβ peptides may be protective against AD in certain situations. In addition, some pathogens directly infect brain tissues, destroying neurons and BBB and further accelerating the pathological process of AD (Piekut et al., 2022). In conclusion, the role of infections in AD is being increasingly scrutinized and studied. Further research in this field not only is beneficial in better understanding the complex etiology of AD but also provides new ideas for the prevention and treatment of AD.

Although inflammation is a well-recognized mechanism linking infections to AD, epigenetic modifications have also been recently highlighted to play a vital role in pathological processes by regulating gene expression (Cummings et al., 2023). Among these modifications, RNA methylation, particularly N6-methyladenosine (m^6^A) and RNA 5-methylcytosine (m^5^C), has emerged as an area of intense interest (Li et al., 2022; PerezGrovas-Saltijeral et al., 2023; Yin et al., 2023; Knight et al., 2024). Emerging evidence underscores that m^5^C methylation, though less studied, is critical in various pathogen infections (Estibariz et al., 2019; Eckwahl et al., 2020; Jia et al., 2021; Jiang et al., 2023). Considering the growing interest in RNA modifications and their potential role in neurodegenerative diseases, we chose to discuss m^5^C methylation in the context of AD. As one of the major post-transcriptional RNA modifications, m^5^C methylation has gradually attracted extensive attention in recent years. Reportedly, m^5^C methylation is involved in regulating diverse biological processes, including splicing, nuclear export, stability, and translation of RNA and biogenesis of microRNAs (miRNAs), further affecting cell differentiation, embryonic development, spermatogenesis, sex determination, learning and memory, cancer onset and progression, and replication and dissemination of pathogenic microbes (Zhao et al., 2017; Bohnsack et al., 2019; Huang et al., 2021b; Wang et al., 2023a; Wang et al., 2023b; Feng et al., 2023; Yan et al., 2023; Xiong et al., 2024). Moreover, recent studies have unveiled those pathogenic microbial infections, as an external factor, affect epigenetic modifications including m^5^C methylation. For example, pathogenic microbial infections change the level of m^5^C methylation in host cells, such as hepatocytes infected with hepatitis viruses (Feng et al., 2023; Chen et al., 2024; Ding et al., 2024). The level of m^5^C RNA methylation plays a crucial role in the anti-infective immune response of the host (Cui et al., 2022; Yu et al., 2022; Chen et al., 2023).

In recent years, researchers have begun to explore the role of RNA methylation in AD and have found the important role of m^5^C methylation in the pathology of AD (PerezGrovas-Saltijeral et al., 2023), which provides novel perspectives for understanding the pathogenesis of AD and presents the possibility of developing new therapeutic strategies for AD.

Overall, m^5^C methylation is emerging as a hotspot in neuroscience and disease research as research deepens. Therefore, this review deeply analyzes the influence of pathogenic microbes on the development of AD and the link between m^5^C methylation and pathogenic microbes, providing a promising research direction for mechanisms underlying infectious AD.

## 2 Risk, prevention, and treatment of AD

As the leading cause of dementia, AD is a progressive neurodegenerative disease mainly characterized by cognitive dysfunction, memory loss, language impairment, and behavioral disorders (Scheltens et al., 2021). According to an updated report on the epidemiology of AD released by the World Health Organization in 2013, approximately 35.6 million people worldwide suffered from AD in 2010, and the number of people living with dementia is expected to triple by 2050 to about 115 million worldwide. Additionally, the prevalence of dementia increases significantly with age. Concretely, the prevalence of AD is 5%–8% among people over the age of 65 years and rises to 25–50% in older people aged above 85 years. Likewise, there is a gender difference in the prevalence of AD, and the prevalence rate is 19–29% lower in men than in women (Khan et al., 2020; Scheltens et al., 2021).

The main factors associated with AD can be categorized into several major types, such as genetic factors, disease factors, poor lifestyle, psychological and mental status, and nutritional status, among which hypertension, sleep disorders, and cardiovascular disease are critical risk factors for the development of AD (Armstrong, 2019; Silva et al., 2019; Zhang et al., 2021). Cardiometabolic and genetic risk factors assume a significant role in the occurrence and progression of AD (Malik et al., 2018). Cardiometabolic risk factors include diabetes mellitus, mid-life obesity, mid-life hypertension, and hypercholesterolemia, which have been associated with cognitive decline in AD patients (Pasqualetti et al., 2022). Prior studies have reported that increased high-density lipoprotein cholesterol concentrations and high systolic blood pressure are associated with a higher risk of developing AD (Malik et al., 2018; Xicota et al., 2019; Luo et al., 2023). In addition, diet is an important nonpharmacological risk-modifying factor for AD (McGrattan et al., 2019; Rusek et al., 2019; Katonova et al., 2022; Stefaniak et al., 2022). Ecological research has revealed that fat and meat from high-energy diets and resultant obesity are key risk factors for AD and that the incidence of AD peaks 15–20 years after consumption of high-energy diets (Grant and Blake, 2023). An observational study assessed the risk of AD in individuals with different dietary patterns and unraveled that the risk of AD was increased by the higher intake of saturated and total fats, meat, and ultra-processed foods but was reduced by the higher intake of fruits, legumes, nuts, and vegetables (Grant and Blake, 2023). Therefore, light diets of low-animal products, as well as low-glycemic load foods, may be effective in preventing AD.

Although anti-amyloid therapies have yielded some favorable results in recent years, it remains highly indispensable to develop comprehensive strategies for combating neurodegenerative diseases. While some preliminary studies have investigated the effects of various compounds and treatments on AD, these findings must be approached with caution. For example, Azizi et al. that carvacrol elevated cell viability, repressed oxidative stress, and ameliorated memory impairment in AD. It is important to emphasize, however, that these findings are obtained based on early-stage research, primarily in cell and animal models of AD, hinting that further studies, particularly clinical trials, are warranted to validate the therapeutic potential of carvacrol for AD (Azizi et al., 2020). Likewise, type 2 diabetes mellitus is tightly correlated with the pathobiology of AD. Some basic science research and clinical trials have elucidated that certain antidiabetic drugs, such as insulin, metformin, and glucagon-like peptide-1 agonists, may reduce the risk of developing AD (Diniz Pereira et al., 2021; Li et al., 2021; Takeishi et al., 2021; Zheng et al., 2021; Du et al., 2022; Kopp et al., 2022). Nonetheless, current evidence is derived largely from hypothetical papers or studies in mouse and cell models. Due to the lack of robust clinical evidence, it is premature to draw definitive conclusions about the efficacy of these antidiabetic drugs in the prevention and treatment of AD.

In summary, the epidemiologic features of AD are complex and diverse and influenced by many factors. It is essential to continuously and intensively investigate the etiology and risk factors of AD and cautiously explore new treatment and prevention methods for AD, thereby addressing this serious public health challenge more effectively in the future.

## 3 Pathogenic mechanisms underlying pathogen infection-induced AD

AD is primarily pathologically characterized by the abnormal deposition of Aβ protein and the neurofibrillary tangles of Tau protein in the brain. Aβ protein deposition disrupts dynamic homeostasis, provoking neuronal death and inflammatory responses. Tau protein hyperphosphorylation leads to the formation of neurofibrillary tangles, compromising cell structure and function. Abnormal changes in Aβ and Tau proteins are essential features of AD, and their interaction triggers intra- and extracellular cascade reactions. Accordingly, investigation into this process is critical for understanding the treatment and diagnosis of AD (Brody, 2011).

### 3.1 Pathological changes in viral infection-induced AD

SARS-CoV-2 enters the central nervous system (CNS) through multiple routes, including blood-borne transmission and trans-synaptic transport, affecting BBB integrity and then causing a bewildering array of neurological symptoms (including headache, olfactory loss, and dysgeusia) and even severe neurological diseases (such as corticospinal tract lesions, Guillain-Barre syndrome, ischemic stroke, encephalopathy, and meningoencephalitis) (Shehata et al., 2021; Baranova et al., 2023). This infectious ability of SARS-CoV-2 is contingent upon the mechanism that it utilizes angiotensin-converting enzyme 2 (ACE2) and transmembrane protease serine 2 (TMPRSS2) to enter host cells (Ata et al., 2023). Notably, ACE2 upregulation is associated with disease progression in AD patients. In addition to ACE2 and TMPRSS2, other host proteins such as neuropilin 1 (NRP1) and dipeptidyl peptidase 4 (DPP4) also serve as potential targets of SARS-CoV-2 in relation to the pathogenesis of AD (Ding et al., 2020; Wicik et al., 2020; Lim et al., 2021). SARS-CoV-2-induced coronavirus disease 2019 (COVID-19) has an impact on the selection of therapies for AD patients. For example, ACE2 activators and Ang II receptor blockers increase the risk of SARS-CoV-2 infections, and certain medications such as donepezil and galantamine elevate the activity of SARS-CoV-2 when used concomitantly with drugs for COVID-19 (Piekut et al., 2022; Edmiston et al., 2023; Le et al., 2024). The COVID-19 pandemic negatively affects the cognitive exercise and mental status of AD patients, particularly in specific populations (Joo et al., 2022). These findings implicate SARS-CoV-2 infections in the pathogenesis of AD. Nonetheless, further studies are required to determine the exact effects and mechanisms of SARS-CoV-2 infections in AD.

Viruses such as HSV have been discovered in the brain tissues of AD patients, especially HSV-1 in Aβ plaques (Piacentini et al., 2014; Mancuso et al., 2019; Zhu and Viejo-Borbolla, 2021). These viruses elevate Aβ levels by interfering with Aβ protein metabolism and disturb the normal function of neurons, partaking in the pathogenesis of AD. HSV-1 also contributes to tau protein hyperphosphorylation, further accentuating neurodegenerative changes (Sait et al., 2021). In addition, the immune response to HSV also potentiates neurodegenerative changes (Mancuso et al., 2020).

Viruses such as cytomegalovirus and varicella zoster virus are also implicated in the development of AD by mediating insulin-degrading enzyme activity or inducing inflammatory responses (de Tullio et al., 2008; Holtappels et al., 2016; Cairns et al., 2022; Mody et al., 2023). hepatitis C virus infections are associated with AD due to the neurotoxic or inflammatory effects of the virus. Specifically, hepatitis C viruses evoke neurodegenerative changes by directly exerting toxic effects on nervous tissues or inducing CNS or systemic inflammation (Vos et al., 2013; Frölich et al., 2017; Huang et al., 2022). Certain hemagglutinins in influenza A viruses participate in the development of AD possibly by enhancing neuroinflammatory and degenerative changes through interactions with Aβ_42_ (Weksler et al., 2002).

Collectively, these viruses are engaged in the pathogenesis of AD via a range of mechanisms, including direct toxic effects on nervous tissues, impact on Aβ and tau protein metabolism, and induction of inflammatory responses. However, further studies are needed to identify the exact association of these viruses with AD and the related mechanisms.

### 3.2 Pathological changes in prokaryotic infection-induced AD

The relationship between prokaryotes and AD is a research area of high interest. Spirochetes are a group of Gram-negative bacteria and include *B. burgdorferi* and *T. pallidum* (Brorson et al., 2009). These bacteria enter the CNS through many routes, leading to infections and latent infections. *B. burgdorferi* and *T. Pallidum* have been detected in the cerebral cortex of AD patients (MacDonald, 2006; Miklossy, 2011; Luo et al., 2015; Herrera-Landero et al., 2019; Senejani et al., 2022). Yet, their relationship with AD is uncertain, which calls for additional research. As a Gram-negative intracellular pathogen associated with CNS infections, *C. pneumoniae* has been found in the brain tissues of AD patients, which contributes to neuroinflammation and is associated with the pathology of AD (Shima et al., 2010; Woods et al., 2020). However, the exact mechanism of *C. pneumoniae* in AD remains under investigation. The oral cavity is an important reservoir of microbes, and oral bacterial dysbiosis leads to diseases of distal organs. Periodontal disease is a common oral disease that is associated with AD. AD patients with periodontal disease present with higher levels of inflammatory responses, which in turn impedes the function of the nervous system. Moreover, specific oral bacteria, such as *C. acnes*, are also associated with the risk of AD (Kornhuber, 1996; Moné et al., 2023). *H. pylori* is a ubiquitous gastrointestinal bacterium, with certain associations with AD as well. Prior studies revealed that the level of anti-*H. pylori* antibodies was higher in AD patients and that *H. pylori* infections resulted in neuroinflammation, contributing to AD pathology (Santos et al., 2020; Xie et al., 2023). Altogether, the relationship between prokaryotes and AD is intricate and diverse. Prokaryotes impair the nervous system through multiple pathways, playing a role in the pathogenesis of AD. However, additional studies are required to ascertain the exact link between prokaryotes and AD and their mechanism in AD.

### 3.3 Pathological changes in eukaryotic infection-induced AD

As a eukaryote, *Toxoplasma gondii* is associated with AD (Nayeri et al., 2021). As reported, long-term exposure to Toxoplasma gondii enhances the risk of neurodegenerative diseases such as AD, as well as other psychiatric disorders including schizophrenia, migraine, and affective disorders. *T. gondii* infections are responsible for numerous neurobiological and behavioral changes, including synaptic loss, decreased nerve fiber density, and behavioral alterations such as anxiety and memory impairment (Nayeri et al., 2021). *T. gondii* adversely affects neurological function and causes AD-like symptoms via a myriad of mechanisms, including interference with the transmission of neurotransmitters such as glutamate, dopamine, and gamma-aminobutyric acid (GABA) (Jung et al., 2012). To be specific, *T. gondii* infections impair the function of N-methyl-D-aspartate receptors, eliciting disturbances in the glutamatergic neurotransmitter system, which in turn compromises neurotransmission and synaptic plasticity (Lucchese, 2017; Lang et al., 2018). In addition, *T. gondii* infections abnormally alter dopamine levels by affecting the dopamine system, thereby interrupting motor control and cognitive function. Meanwhile, abnormalities in the GABAergic system are also implicated in cognitive decline (Brooks et al., 2015). Nevertheless, although *T. gondii* infections are correlated with AD, they have not been observed to promote the aggregation of pathological proteins such as Aβ and tau. Instead, some studies have elucidated that chronic toxoplasmosis reduces the burden of Aβ plaques and functions as a potential protective factor against cognitive decline (Möhle et al., 2016), which is achieved by modulating the levels of anti-inflammatory cytokines. To summarize, *T. gondii* infections impede neurological function through several pathways, participating in the pathogenesis of AD. Nonetheless, further research is still required to reveal the specific mechanisms underlying the complex relationship between *T. gondii* infections and AD and develop possible therapeutic and prevention strategies for AD.

## 4 Effects of infections on m^5^C RNA methylation

The discovery of m^5^C RNA methylation can be dated back to the 1950s, predating the discovery of the double helix structure of DNA. m^5^C, a key RNA methylation modification, has emerged as a hot research topic in recent years. Because of the development of methylation sequencing technology, massive m^5^C methylation has been identified in both coding and non-coding RNAs. m^5^C RNA methylation is regulated by methyltransferases, demethylases, and m^5^C-binding proteins, which modulates RNA stability, translocation, translation, and stress and is involved in tumor development, pathogenic microbial replication and dissemination, and the biological functions of immunomodulation (Zhao et al., 2017; Bohnsack et al., 2019).

### 4.1 Basic features of m^5^C RNA methylation

m^5^C methylation is formed by adding an active methyl group from the donor, usually S-adenosyl-methionine, to the carbon-5 position of the cytosine base in RNA (Zhao et al., 2017), which is an RNA modification widely present in messenger RNA (mRNA) and non-coding RNAs including transfer RNA (tRNA), ribosomal RNA, long non-coding RNA, small nuclear RNA, miRNA, and enhancer RNA. The distribution of m^5^C methylation varies across species. For instance, m^5^C methylation is more in eukaryotic tRNA and mRNA than in bacterial mRNA and tRNA (Song et al., 2022).

m^5^C methylation is found in both the nucleus and cytoplasm. In the nucleus, m^5^C methylation primarily occurs in mRNA, tRNA, and rRNA, where it modulates RNA stability, splicing, and export. In the cytoplasm, m^5^C methylation is mainly present in tRNA and mRNA, where it influences translation and RNA stability (Zhao et al., 2017; Bohnsack et al., 2019). m^5^C methylation is mainly mediated by three classes of proteins: methyltransferases (writers), demethylases (erasers), and m^5^C-binding proteins (readers). Methyltransferases (writers) consist of DNA methyltransferase 2 (DNMT2), tRNA-specific methyltransferase (TRDMT) family members, and NOL1/NOP2/SUN domain (NSUN) family members (NSUN1-7 and NSUN5a/b/c) and utilize adenosylmethionine as a methyl donor to form m^5^C by transferring the methyl group to a cytosine (Bohnsack et al., 2019). Enzymes in the NSUN and DNMT families contain conserved motifs IV and VI, possess complementary target specificities, and catalyze cytosine-5 methylation (Xu et al., 2010). Demethylases (erasers), including enzymes in the ten-eleven translocation (TET) family (such as TET1, TET2, and TET3), oxidize m^5^C to exert a reversible effect, therefore mediating RNA demethylation. TET1 can oxidize 5-formylcytosine to 5-carboxycytosine in RNA, and TET2 can inhibit the effect of 5-methylcytosine on double-stranded RNA formation (Shen et al., 2021; Yang et al., 2022; Li et al., 2023; Lin et al., 2024). Additionally, ALKBH1 is responsible for the demethylation of tRNA (Chen et al., 2021). m^5^C-binding proteins (readers), such as Aly/REF export factor (ALYREF) and Y-box binding protein 1 (YBX1), exert biological effects by recognizing and binding to m^5^C sites. ALYREF recognizes m^5^C in RNA and contributes to the export of RNA to the cytoplasm (Yang et al., 2017). YBX1 is an m^5^C-reading protein that specifically targets cytoplasmic mRNA and increases its stability (Li et al., 2024).

Comparatively, m^6^A methylation is the most ubiquitous internal modification in eukaryotic mRNA, which has been extensively studied. m^6^A is added by methyltransferase complexes containing METTL3 and METTL14 (writers), removed by demethylases such as FTO and ALKBH5 (erasers), and recognized by reader proteins such as YTH domain family proteins (Zhao et al., 2017; Jiang et al., 2021a; An and Duan, 2022). Both m^5^C and m^6^A modifications mediate RNA stability, splicing, export, and translation (Zhao et al., 2017). However, m^6^A primarily regulates mRNA metabolism and assumes a pivotal role in processes including stem cell differentiation, circadian rhythm, and stress responses (Zhao et al., 2017), whereas m^5^C is involved in a broader range of RNA species and has distinct roles in tRNA function and RNA transport, hinting at the unique regulatory capacity of each modification (Bohnsack et al., 2019; Delaunay et al., 2022). These features of m^5^C methylation shed light on the importance of m^5^C RNA methylation in the regulation of gene expression and cellular functions, as well as its diverse roles in different biological processes.

### 4.2 Relationship between m^5^C methylation and pathogen infections

Viral infections have diverse and intricate effects on m^5^C RNA methylation. Infections with Zika virus and HSV markedly reduce m^5^C methylation levels in host cells, which contributes to defenses against viral infections, thereby inhibiting viral infections and replication (Wang et al., 2023c). Hepatitis B virus infections significantly affect the distribution of m^5^C methylation in human hepatocytes, suggesting that the effect of infections may be related to the type of host cells (Feng et al., 2023; Chen et al., 2024; Ding et al., 2024). A prior study showed that SARS-CoV-2 infections prominently reduced NSUN2 mRNA levels in host cells (Wang et al., 2023c). In viral infections, NSUN2 assumes a pivotal role in regulating m^5^C methylation, influencing gene expression and viral replication. For example, NSUN2 mediates m^5^C methylation, affecting viral replication in murine leukemia virus infections (Eckwahl et al., 2020). In human immunodeficiency virus-1 infections, NSUN2 modulates m^5^C RNA methylation to impact multiple stages of viral replication (Courtney et al., 2019; Winans and Beemon, 2019). Additionally, viruses such as flavivirus, hepatitis C virus, and Zika virus are also orchestrated by NSUN2 (Hagist et al., 2009; Wang et al., 2023c). Furthermore, Sinefungin and its related metabolite A9145C are competitive inhibitors of S-adenosine-L-methionine-dependent enzymes with much lower inhibition constants than S-adenosine-l-homocysteine, which represses the replication of dengue and Zika viruses (Pugh et al., 1978; Wnuk et al., 2020). Malaria is a parasitic disease attributed to plasmodium infections and, together with acquired immunodeficiency syndrome and tuberculosis, constitutes the three major global infectious diseases. Of note, malaria is endemic in nearly 90 countries and territories worldwide (Savi, 2022). Through the functional clustering analysis of m^5^C target genes, a prior study demonstrated that m^5^C methylation was involved in the sexual reproduction process of plasmodium and that NSUN2 was a key methyltransferase responsible for m^5^C RNA methylation in plasmodium. In addition, this study also exhibited that NSUN2 deletion directly lowered the level of m^5^C methylation in target gene transcripts related to the development of plasmodium gametophytes, drastically reducing the ability of plasmodium to produce mature gametophytes, and that infections with NSUN2-knockout parasites also substantially decreased the number of plasmodium at all stages of sexual development, ultimately suppressing malaria transmission (Liu et al., 2022).

The effects of infections on m^5^C RNA methylation levels are diverse and complex, depending on the type of the pathogen and host cell or tissue involved. In the context of AD, m^5^C methylation can be altered in cells in the CNS (such as neurons and glial cells) and circulating immune cells, potentially impacting both direct and indirect pathways involved in the pathogenesis of AD. Hence, future studies should focus on the complex interactions between infections and RNA modifications, providing novel ideas and directions for research on the mechanisms of viral infections and the development of new therapeutic strategies for AD.

### 4.3 Role of m^5^C RNA methylation in immunomodulation

m^5^C RNA methylation is essential for the host immune response against infections (Cui et al., 2022; Yu et al., 2022; Chen et al., 2023). mRNA methylation orchestrates protein expression in dendritic cells. Dendritic cells are activated when exposed to non-self components. Dendritic cell activation can be stimulated by RNA transcribed *in vitro* (such as RNA in mammalian necrotic cells) but can be attenuated or eliminated by RNAs with m^5^C methylation. Hence, higher methylation levels are associated with the stronger repressive effect of methylation on dendritic cell activation (Kariko et al., 2005). Chen et al. discovered that NSUN5-mediated m^5^C methylation of GPX4 activated cGAS-STING signaling in cancer immunotherapy of colon adenocarcinoma (Chen et al., 2023). Zhang et al. observed that in infections with RNA viruses (such as respiratory syncytial virus, vesicular stomatitis virus, human metapneumovirus, and Sendai virus) and DNA viruses (including HSV), NSUN2 diminished the levels of specific non-coding RNAs, particularly RPPH1 and 7SL RNAs, and altered the level of m^5^C methylation, which directly or indirectly regulated type I interferon (IFN) responses mediated by the retinoic acid-inducible gene I pathway and therefore enhanced antiviral responses (Zhang et al., 2022). Another study displayed that NSUN2 specifically orchestrated m^5^C methylation of interferon regulatory factor 3 (IRF3) mRNA and accelerated its degradation, declining the levels of IRF3 and downstream IFN-β, and that knockout or knockdown of NSUN2 increased the production of type I IFN and downstream IFN-stimulated genes during various viral infections *in vitro* (Wang et al., 2023c).

Naive CD4^+^ T cells (Th0 cells) leave the thymus and differentiate into different cell subpopulations, including T helper cells (Th1, Th2, Th9, Th17, and Th22), T follicular helper cells (Tfh), and T regulatory cells (Treg), in response to variable activation signals (Zhu and Zhu, 2020). Several studies have reported that m^5^C methylation modulates the biological processes of CD4^+^ T cells and their multiple subpopulations. For instance, a former study unveiled that m^5^C methylation levels and NSUN2 expression were reduced in CD4^+^ T cells of patients with systemic lupus erythematosus and that m^5^C hypermethylation was closely associated with immune- and inflammation-related diseases, such as systemic lupus erythematosus, via pathways including the immune system, cytokine signaling, and IFN signaling (Guo et al., 2020). Another study revealed that deletion of the m^5^C methyltransferase NSUN2 in mouse CD4^+^ T cells specifically depressed Th17 cell differentiation and relieved Th17 cell-induced colitis (Yang et al., 2023). Although multiple studies have unraveled that m^5^C RNA methylation has an essential role in the function of immune organs and provided new insights into the molecular mechanisms behind immune responses (Wang et al., 2022), little is known about the regulatory mechanisms and targeted therapies of m^5^C RNA methylation in AD. In conclusion, m^5^C RNA methylation plays a key role in regulating host antiviral responses, dendritic cell activation, CD4^+^ T cell differentiation, and immune-related diseases. However, future studies need to further probe the specific mechanisms of m^5^C RNA methylation in AD and its potential applications in the treatment of AD.

## 5 m^5^C methylation in AD

Accumulating evidence unravels that the occurrence of neurocognitive disorders is associated with changes in m^6^A and m^5^C methylation systems. As previously reported, familial mutations in the m^6^A methylation-related gene METTL5 and m^5^C methylation-related genes NSUN2, NSUN3, NSUN5, and NSUN6 are implicated in intellectual developmental disorders. Moreover, these genes form a protein complex with the m^5^C methylation reading protein ALYREF. The expression of m^5^C methylation-related writing and reading proteins NSUN6, NSUN7, and ALYREF varies across individuals with AD, high neuropathological burden, or traumatic brain injury. These findings elucidate that the RNA methylation system may underlie neurocognitive disorders by impairing neural and synaptic function via a series of molecular mechanisms (Jiang et al., 2021b; Deng et al., 2021; Li et al., 2022; Liu et al., 2023; Yin et al., 2023; Knight et al., 2024).

In a prior study, RNA sequencing data of 31 effector proteins from four brain regions of 51 AD patients were analyzed to investigate the role of 5mC/5hmC and m^5^C effector proteins in the neuropathology of AD. Additionally, gene expression profiles were compared between AD patients and control individuals. The results displayed that the expression of RNA methylation-related writers NSUN6 and NSUN7 was significantly different in AD and, along with the expression of the reader ALYREF, different for the neurodegenerative ranking. These results illustrate that in AD, the regulation of protein pathways is disrupted via multiple pre- and post-transcriptional mechanisms, potentially involving tRNAs, enhancer RNAs, nuclear-cytoplasmic shuttling, and cytoplasmic translational control. Accordingly, targeting these processes can open up new avenues for the treatment of neurodegenerative conditions (PerezGrovas-Saltijeral et al., 2023).

In AD, the changed level of m^5^C RNA methylation is believed to be involved in neuropathological alterations, including neuronal and synaptic dysfunction, thus forming the basis of neurocognitive disorders. However, the causes of changes in m^5^C RNA methylation levels are still poorly understood, which calls for more studies on the potential mechanisms in this field.

## 6 m^5^C methylation as a potential mechanism for infectious AD

As a prevalent neurodegenerative disorder, AD has a complex pathogenesis that has not yet been fully elucidated. In recent years, the relationship between pathogen infections and AD has garnered growing attention, leading to the emergence of a new theory known as the infectious etiology of AD, which posits that pathogen infections may promote the onset of AD through various mechanisms. Notably, the infection theory is independent of other mechanisms of AD pathogenesis and may interact with other theories such as the amyloid cascade hypothesis and the tau protein hypothesis, collectively participating in the progression of AD. Additional research is warranted in the future to further explore the specific correlation and mechanisms between pathogen infections and AD, therefore providing new strategies for the prevention and treatment of AD. As summarized above, pathogen infections can result in changes in m^5^C RNA methylation levels in the host, as evidenced by alterations in m^5^C RNA methylation levels in AD patients observed in recent research. The mechanism by which pathogen infections cause the onset and progression of AD is still under investigation, with pathogen infection-m^5^C methylation-AD as a promising research direction in the field of AD. No relevant research results have been reported, necessitating increasing attention to this direction in the future.
